# Supplementary material for: Bioaccessible Raspberry Extracts Enriched in Ellagitannins and Ellagic Acid Derivatives Have Anti-Neuroinflammatory Properties
Source: Antioxidants (Basel). 2020 Oct 10;9(10):970. doi: 10.3390/antiox9100970 (PMC7600793; doi:10.3390/antiox9100970)
Supplement: Supplementary file 1 [file antioxidants-09-00970-s001.pdf]

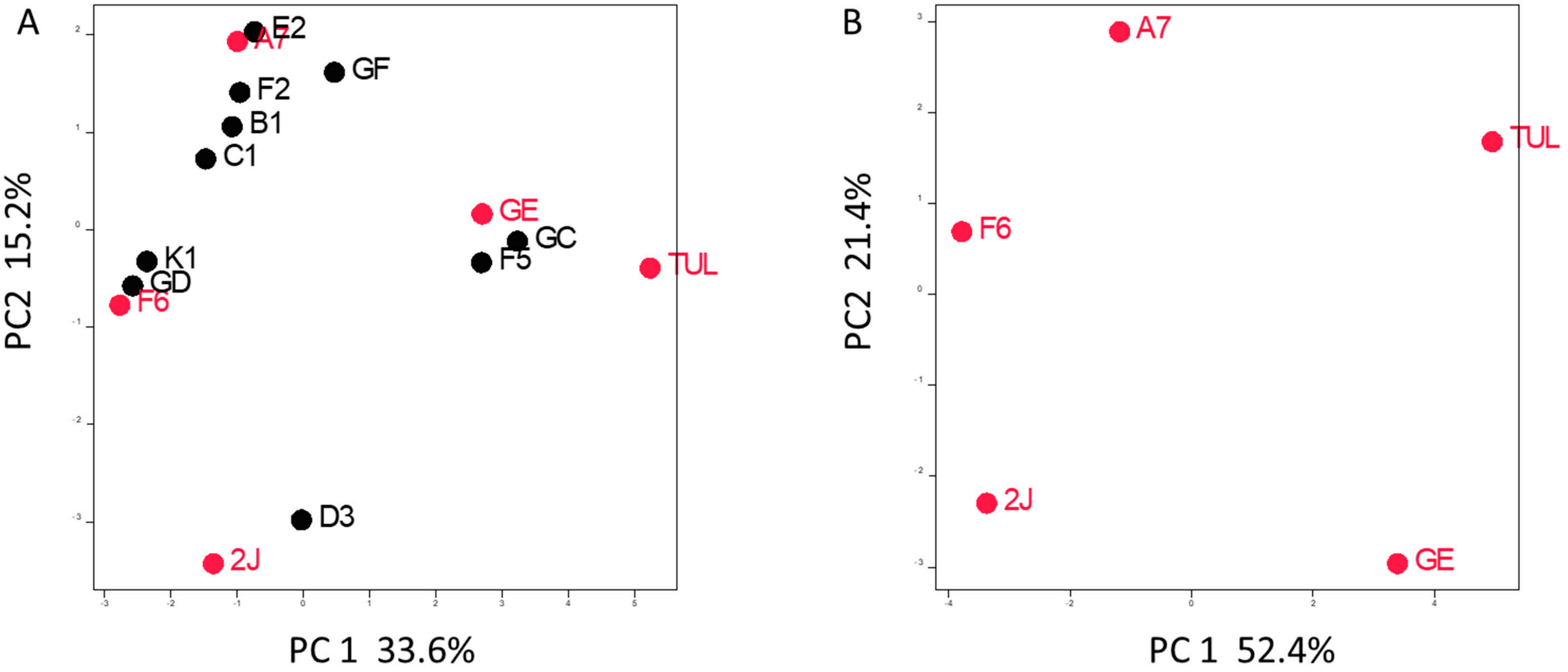


**Supplementary Figure S1. PCA plot of polyphenol composition of: A.** Extracts from raspberry cultivars and genotypes **B.** GIB fractions of selected raspberry cultivars and genotypes. Raspberry cultivars Glen Cally (GC), Glen Doll (GD), Glen Ericht (GE), Glen Fyne (GF), Tulameen (TUL) and genotypes 2J19 (2J), 00123A7 (A7), 0019E2 (E2), 0304F6 (F6), 0435D-3 (D3), 0460F-5 (F5), 0485K-1 (K1), 0534RB-1 (B1) & 9455F-2 (F2), 9911C-1 (C1). Cultivars and genotypes in red were selected for *in vitro* digestion.

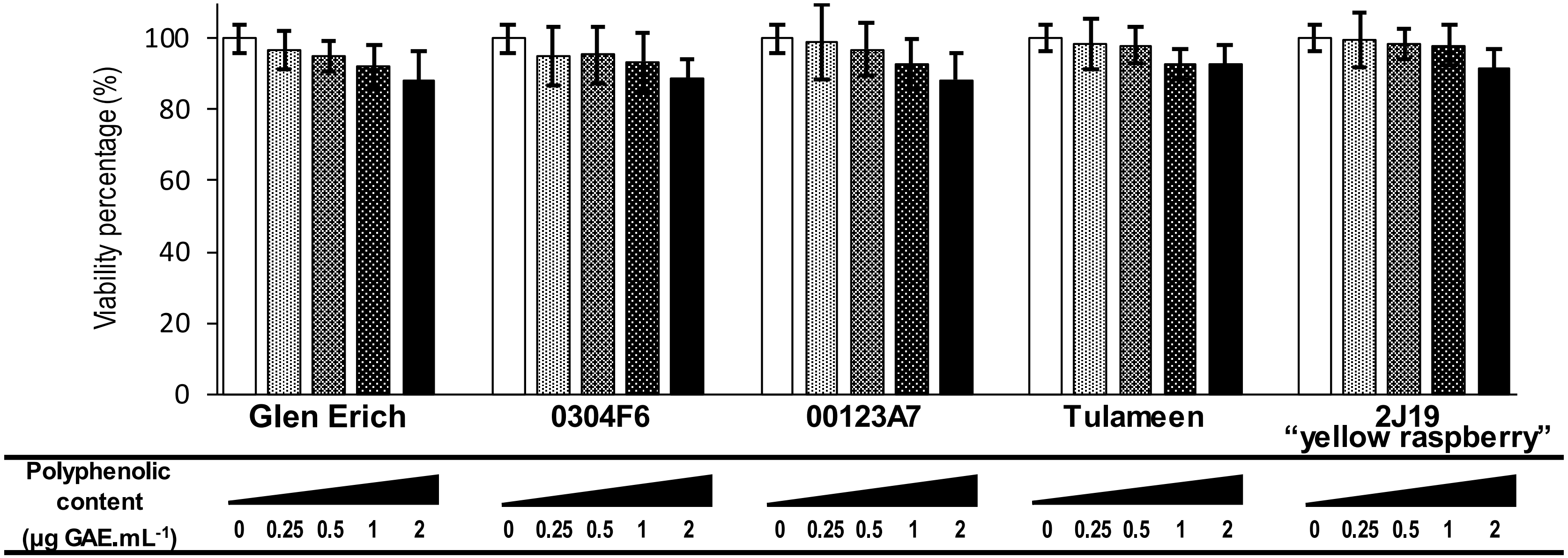


**Supplementary Figure S2. Cytotoxicity of the five raspberry GIB fractions under physiological concentrations.** None of the five GIB fractions demonstrated significant cytotoxic effects at 0.25, 0.5, 1 and 2 µg GAE.mL$^{-1}$, even after 24 h of incubation.

**Supplementary Table S1. Major polyphenols in raspberry cultivars and genotypes**

| Compound | $R_t$ (min) | Glen Cally | Glen Doll | Glen Ericht | Glen Fyne | Tulameen | 00123A7 | 0019E2 | 0304F6 |
|---|---|---|---|---|---|---|---|---|---|
| **Anthocyanin (μg g$^{-1}$ FDW)** | | | | | | | | | |
| Cyanidin 3-sophoroside | 16.36 | 3582.7 | 2781.2 | 3437.9 | 2579.0 | 3671.0 | 1831.8 | 2573.5 | 1213.7 |
| Cyanidin 3-sophoroside-5-rhamnoside | 17.04 | 975.3 | 16.2 | 913.5 | 1245.8 | 1191.8 | 410.8 | 633.2 | 24.3 |
| Cyanidin 3-glucoside | 17.20 | 535.0 | 700.3 | 527.4 | 441.6 | 606.7 | 650.5 | 756.3 | 261.1 |
| Pelargonidin3-sophoroside / cyanidin 3-rutinoside | 17.98 | 499.0 | 187.1 | 511.6 | 507.8 | 558.5 | 212.7 | 325.5 | 68.1 |
| Total | | 5592.0 | 3684.8 | 5390.4 | 4774.1 | 6028.0 | 3105.9 | 4288.5 | 1567.2 |
| **Flavonol (μg g$^{-1}$ FDW)** | | | | | | | | | |
| Quercetin 3-glucosylrutinoside | 20.16 | 40.73 | 2.10 | 40.03 | 37.79 | 46.30 | 12.64 | 22.10 | 0.56 |
| Quercetin diglucoside | 21.02 | 10.15 | 30.78 | 9.54 | 5.75 | 14.21 | 7.15 | 8.37 | 13.55 |
| Quercetin 3-galactoside | 24.11 | 16.11 | 0.00 | 12.93 | 0.00 | 19.85 | 26.90 | 15.50 | 6.12 |
| Quercetin 3-glucuronide | 24.34 | 12.10 | 9.51 | 9.90 | 12.29 | 34.73 | 108.68 | 25.77 | 4.29 |
| Quercetin 3-glucoside | 24.44 | 4.09 | 11.33 | 3.41 | 4.03 | 6.34 | 11.13 | 8.84 | 2.71 |
| Total | | 83.2 | 53.7 | 75.8 | 59.9 | 121.4 | 166.5 | 80.6 | 27.2 |
| **Ellagic acid conjugates (μg g$^{-1}$ FDW)** | | | | | | | | | |
| Ellagic acid pentoside A | 23.15 | 37.66 | 12.50 | 33.60 | 17.69 | 40.72 | 13.24 | 14.16 | 11.25 |
| Ellagic acid pentoside B | 23.64 | 29.71 | 20.52 | 27.35 | 17.55 | 5.48 | 14.41 | 18.62 | 20.13 |
| Ellagic acid | 24.01 | 10.16 | 4.29 | 8.54 | 9.34 | 16.70 | 5.01 | 3.83 | 9.73 |
| Methyl ellagic acid pentoside A | 26.53 | 13.83 | 5.73 | 13.31 | 4.51 | 22.88 | 4.85 | 4.75 | 4.04 |
| Ellagic acid acetylpentoside A | 26.67 | 5.27 | 6.76 | 5.20 | 10.39 | 4.50 | 6.16 | 7.17 | 6.36 |
| Ellagic acid acetylpentoside B | 27.68 | 7.30 | 17.83 | 7.43 | 14.65 | 1.66 | 12.09 | 15.38 | 19.93 |
| Total | | 103.9 | 67.6 | 95.4 | 74.1 | 91.9 | 55.8 | 63.9 | 71.4 |
| **Ellagitannins (relative levels)** | | | | | | | | | |
| Lambertianin C | 21.01 | 100.0 | 45.6 | 86.9 | 55.4 | 48.2 | 85.3 | 98.0 | 39.9 |
| Sanguiin H6 | 21.60 | 100.0 | 77.5 | 89.6 | 81.6 | 87.8 | 77.3 | 81.4 | 70.5 |

| Compound | Rt (min) | 0435D-3 | 0485K-1 | 0534RB-1 | 0460F-5 | 9455F-2 | 9911C-1 | 2J19 |
|---|---|---|---|---|---|---|---|---|
| **Anthocyanin (μg g$^{-1}$ FDW)** | | | | | | | | |
| Cyanidin 3-sophoroside | 16.36 | 3224.8 | 2754.7 | 1696.3 | 4473.1 | 2632.4 | 3210.4 | 20.7 |
| Cyanidin 3-sophoroside-5-rhamnoside | 17.04 | 0.0 | 21.8 | 573.2 | 758.4 | 565.9 | 1.3 | 0.6 |
| Cyanidin 3-glucoside | 17.20 | 534.4 | 459.3 | 465.6 | 819.0 | 618.1 | 805.0 | 18.2 |
| Pelargonidin3-sophoroside / cyanidin 3-rutinoside | 17.98 | 125.1 | 140.9 | 297.1 | 451.6 | 268.9 | 147.8 | 0.0 |
| Total | | 3884.3 | 3376.7 | 3032.1 | 6502.2 | 4085.3 | 4164.4 | 39.5 |
| **Flavonol (μg g$^{-1}$ FDW)** | | | | | | | | |
| Quercetin 3-glucosylrutinoside | 20.16 | 0.00 | 3.30 | 17.86 | 33.58 | 15.45 | 0.00 | 0.00 |
| Quercetin diglucoside | 21.02 | 28.38 | 28.07 | 7.97 | 12.16 | 7.37 | 17.75 | 2.63 |
| Quercetin 3-galactoside | 24.11 | 3.59 | 0.00 | 5.85 | 0.00 | 5.98 | 10.40 | 0.00 |
| Quercetin 3-glucuronide | 24.34 | 4.34 | 26.48 | 1.85 | 20.88 | 3.42 | 6.62 | 24.06 |
| Quercetin 3-glucoside | 24.44 | 5.51 | 6.24 | 1.39 | 5.97 | 2.23 | 3.05 | 13.94 |
| Total | | 41.8 | 64.1 | 34.9 | 72.6 | 34.5 | 37.8 | 40.6 |
| **Ellagic acid conjugates (μg g$^{-1}$ FDW)** | | | | | | | | |
| Ellagic acid pentoside | 23.15 | 28.97 | 19.29 | 17.17 | 32.85 | 18.34 | 18.47 | 25.75 |
| Ellagic acid pentoside | 23.64 | 30.36 | 16.26 | 13.78 | 17.93 | 20.62 | 16.70 | 20.05 |
| Ellagic acid | 24.01 | 8.39 | 6.43 | 7.21 | 7.03 | 5.09 | 6.30 | 10.08 |
| Methyl ellagic acid pentoside | 26.53 | 10.81 | 5.15 | 6.57 | 16.64 | 6.23 | 4.61 | 13.96 |
| Ellagic acid 4-acetylxyloside | 26.67 | 2.96 | 11.17 | 11.75 | 2.59 | 8.29 | 8.64 | 5.77 |
| Ellagic acid 4-acetylarabinoside | 27.68 | 5.32 | 16.46 | 13.44 | 2.21 | 15.89 | 11.92 | 6.49 |
| Total | | 86.8 | 74.8 | 69.9 | 79.3 | 74.5 | 66.6 | 82.1 |
| **Ellagitannins (relative levels)** | | | | | | | | |
| Lambertianin C | 21.01 | 60.2 | 65.8 | 40.4 | 13.9 | 93.6 | 89.0 | 61.9 |
| Sanguiin H6 | 21.60 | 97.4 | 88.0 | 75.3 | 57.7 | 75.4 | 82.0 | 86.0 |

No standards were available for quantification of the major ellagitannins, so they were expressed as % levels relative to Glen Cally which had the highest levels.

## Supplementary Table S2. Detailed polyphenol composition of the selected raspberry genotypes

| Compound | $R_t$ (min) | $[M+H]^+$ (*m/z*) | | $MS^2$ (*m/z*) | 00123A7 | 0304F6 | 2J19 | Glen Ericht | Tulameen |
|---|---|---|---|---|---|---|---|---|---|
| **Anthocyanins ($\mu g\ g^{-1}$ FDW)** | | Observed | Actual | | | | | | |
| Cyanidin 3,5-diglucoside | 13.55 | 611.1614 | 611.1612 | 449, **287** | 14.4±0.6b | 15.5±2.3b | 0±0a | 58.4±14.7d | 22.6±2.1c |
| Cyanidin 3-sophoroside | 16.27 | 611.1609 | 611.1612 | 287 | 1723.3±88.3c | 936.8±72b | 12.7±0.8a | 3482.5±986.1d | 3509.3±174d |
| Cyanidin 3-sophoroside-5-rhamnoside | 16.86 | 757.2195 | 757.2191 | 611, 433, **287** | 317.5±15.2 c | 16.7±1.3b | 0.1±0.1a | 609.2±110.1d | 771.3±23e |
| Cyanidin 3-glucoside | 17.06 | 449.1082 | 449.1084 | 287 | 637.1±36.6d | 229.1±18.5b | 9±0.9a | 435.4±93.5c | 491.1±18.3c |
| Cyanidin 3-sambubioside | 17.33 | 581.1510 | 581.1507 | 287 | 63.9±2.6e | 49.6±3.3d | 0±0a | 28.5±6b | 36.3±1.5c |
| Pelargonidin 3-sophoroside / Cyanidin 3-rutinoside | 17.47 | 595.1669 | 595.1663 | 449, 271 / **287** | 180.3±7.3c | 49.5±3.4b | 0.4±0a | 369.2±71.1d | 392.8±20.4d |
| Cyanidin 3-sambubioside-5-rhamnoside | 17.72 | 727.2093 | 727.2086 | 581, 433, **287** | 13.9±0.5cd | 12.7±1c | 0±0a | 8.7±1.5b | 15.5±0.8d |
| Pelargonidin 3-sophoroside-5-rhamnoside | 18.07 | 741.2252 | 741.2242 | 595, 417, **271** | 12.2±0.4c | 2.1±0.2b | 0±0a | 52.3±9.4d | 51.8±2.6d |
| Pelargonidin 3-glucoside | 18.37 | 433.1133 | 433.1135 | 271 | 34.6±2.3d | 10.3±0.5b | 0.1±0a | 49.8±11e | 26.1±1.3c |
| Pelargonidin 3-sambubioside | 18.94 | 565.1559 | 565.1557 | ND | 1.7±0.1c | 2±0.1c | 0±0a | 1.3±0.3b | 1.3±0b |
| Pelargonidin 3-rutinoside | 19.12 | 579.1714 | 579.1714 | 433, **271** | 3.5±0.3c | 1.2±0b | 0±0a | 17.5±3.5e | 10.5±0.4d |
| **Total Anthocyanins** | | | | | **3002.5±152.2c** | **1325.4±100.8b** | **22.4±1.7a** | **5112.7±1303.5d** | **5328.6±237.7d** |
| **Flavonols ($\mu g\ g^{-1}$ FDW)** | | **$[M-H]^-$ (*m/z*)** | | | | | | | |
| Quercetin 3-glucosylrutinoside | 20.33 | 771.2001 | 771.1984 | 591, **301** | 8.64±1.3c | 0.5±0.03b | 0±0a | 24.67±19.23c | 37.71±27.21c |
| Quercetin diglucoside | 21.26 | 625.1414 | 625.1405 | 300 | 3.44±0.28b | 10.54±0.91d | 1.17±0.26a | 6.24±1.89c | 9.14±4.09cd |
| Quercetin 3-galactosylrhamnoside*[1] | 23.81 | 609.1473 | 609.1456 | 301 | 2.46±0.22bc | 0.01±0a | 0.01±0.02a | 1±0.47b | 4.8±2.12c |
| Quercetin 3-rutinoside*[1] | 24.04 | 609.1469 | 609.1456 | 301 | 3.31±0.28c | 0.64±0.04b | 0.02±0.01a | 2.95±1.67c | 4±1.94c |
| Quercetin 3-galactoside | 24.53 | 463.0893 | 463.0877 | 301 | 32.44±7.56d | 6.28±0.79b | 0±0A | 11.47±1.63c | 26.94±1.01d |
| Quercetin 3-glucuronide | 24.82 | 477.0684 | 477.0669 | 301 | 137.04±22.14e | 4.28±0.65a | 26.91±8.05C | 7.52±1.4b | 39.53±4.43d |
| Quercetin 3-glucoside | 24.87 | 463.0891 | 463.0877 | 301 | 8.12±0.43c | 2.32±0.16a | 12.98±2.38D | 2.46±0.32a | 4.67±0.23b |
| Quercetin HMGG*[2] | 26.11 | 607.1311 | 607.1299 | 545, 505, **463**, 301 | 0.36±0.05c | 0±0a | 0±0A | 0.05±0.01b | 16.69±1.12d |
| Kaempferol 3-galactoside | 26.26 | 447.0944 | 447.0927 | 285 | 1.43±0.25c | 0.29±0.06b | 0±0A | 1.79±0.18cd | 2.07±0.14d |
| Kaempferol 3-glucuronide | 27.17 | 461.0736 | 461.0720 | 285 | 7.51±1.52e | 0.24±0.05a | 0.94±0.19B | 1.45±0.2c | 2.79±0.32d |
| Kaempferol 3-glucoside | 27.08 | 447.0940 | 447.0927 | | 1.33±0.2d | 0.15±0.01a | 0.36±0.03B | 0.56±0.06c | 1.22±0.08d |
| **Total Flavonols** | | | | | **206.06±31.02c** | **25.26±2.43a** | **42.37±10.91B** | **60.17±25.99b** | **149.57±39.2c** |
| **Compound** | **$R_t$ (min)** | **$[M+H]^+$ (*m/z*)** | | **$MS^2$ (*m/z*)** | **00123A7** | **0304F6** | **2J19** | **Glen Ericht** | **Tulameen** |
| **Ellagic acid derivatives ($\mu g\ g^{-1}$ FDW)** | | **$[M-H]^-$ (*m/z*)** | | | | | | | |
| Ellagic acid pentoside A | 23.00 | 433.0420 | 433.0407 | 301 | 12.2±1.2a | 8.5±0.3a | 23.4±4.6B | 24.3±9.2b | 35.1±7.3b |
| Ellagic acid pentoside B | 23.59 | 433.0422 | 433.0407 | 301 | 13.4±1.7b | 18.4±0.6cd | 16.1±1.6bc | 22.2±3.3d | 4.5±0.4a |

| | | | | | | | | | | | | | | |
|---|---|---|---|---|---|---|---|---|---|---|---|---|---|---|
| Ellagic acid | 24.22 | 300.9996 | 300.9984 | | | 7.4±1.7a | | 8.9±0.4ab | | 10.6±0.2B | | 9.9±1.6b | | 19.7±3.8c |
| Methyl ellagic acid pentoside A | 26.58 | 447.0579 | 447.0564 | 315 | | 4.1±0.5a | | 3.4±0.1a | | 11.6±2C | | 8.5±2.4b | | 17±2.4d |
| Ellagic acid acetylxyloside*[3] | 26.99 | 475..0528 | 475.0513 | 301 | | 6.6±1.3b | | 5.8±0.7b | | 5.9±0.1B | | 4.2±0.5a | | 4.4±1a |
| Methyl ellagic acid pentoside B | 27.32 | 447.0578 | 447.0564 | 315 | | 1±0.2b | | 1.7±0.1c | | 2.9±0.3D | | 2±0.3c | | 0.6±0a |
| Ellagic acid acetylarabinoside*[3] | 28.11 | 475.0525 | 475.0513 | 300 | | 14.6±3.6c | | 20.6±2.3d | | 6.4±0.4B | | 6.8±1.4b | | 1.8±0.4a |
| **Total EA derivatives** | | | | | | **59.3±1.5a** | | **67.3±0.6ab** | | **76.9±1.3b** | | **77.9±2.7b** | | **83.1±2.2b** |
| **Ellagitannins (relative levels)** | | **[M-H]$^{-2}$ *(m/z)*** | | | | | | | | | | | | |
| Lambertianin C | 21.46 | 1401.1081*[3] | 1401.1069 | 1401 | | 100.0±2.2a | | 89.1±3.5a | | 84.5±10.1a | | 99.5±11.2a | | 87.3±7.3a |
| Sanguiin H6 | 22.07 | 934.0729*[3] | 934.0712 | 1567, 1235 633, 301 | | 95.7±3.2a | | 92.1±3.4b | | 91.83±9.1a | | 99.2±10.6a | | 100.0±6.0a |
| **Total Ellagitannins*[4]** | | | | | | **195.7a** | | **181.2b** | | **176.3a** | | **198.5a** | | **187.3a** |
| **Total Phenols*[5] (mg g$^{-1}$ FDW)** | | | | | | **44124a** | | **33924b** | | **58512c** | | **96024d** | | **52704c** |

Values are ug g FDW$^{-1}$ ± standard deviation, except for ellagitannins. Values with the same letter were not significantly different $p<0.05$.

*[1] Quercetin 3-galactosylrhamnoside and quercetin 3-rutinoiside have identical mass spectra and were tentatively assigned by elution order (Mullen et al., 2003). *[2] Quercetin 3-O-(3-hydroxy-3-methyl-glutaroyl)-galactoside. *[3] Quercetin acetylpentosides putatively identified by elution order (Mullen et al., 2003).

*[3] Doubly charged ions.

*[4] No standards were available for quantification of the major ellagitannins, so their peak areas were expressed as % relative to the Lambertianin C peak which had the highest levels. Therefore, total values are only indications of relative content. This explains why the total Ellagitannins value are >100%.

*[5] Assayed by Folin method

**Supplementary Table S3A. Recovery of polyphenols in raspberry GIB fractions**.

| Compound | | 00123A7 | | 0304F6 | | 2J19 | | Glen Ericht | | Tulameen | |
|---|---|---|---|---|---|---|---|---|---|---|---|
| | | µg g$^{-1}$ FDW | %Rec | µg g$^{-1}$ FDW | %Rec | µg g$^{-1}$ FDW | %Rec | µg g$^{-1}$ FDW | %Rec | µg g$^{-1}$ FDW | %Rec |
| **Anthocyanins** | Cyanidin 3,5-diglucoside | 1.4 | **9.6** | 0.9 | **6.0** | 0.0 | **0.0** | 11.7 | **20.1** | 1.3 | **5.7** |
| | Cyanidin 3-sophoroside | 186.2 | **10.8** | 126.6 | **13.5** | 1.2 | **9.8** | 381.3 | **11.0** | 434.9 | **12.4** |
| | Cyanidin 3-sophoroside-5-rhamnoside | 26.0 | **8.2** | 1.4 | **8.1** | 0.0 | **0.0** | 83.5 | **13.7** | 111.8 | **14.5** |
| | Cyanidin 3-glucoside | 44.4 | **7.0** | 17.5 | **7.6** | 2.7 | **30.1** | 34.2 | **7.8** | 39.1 | **8.0** |
| | Cyanidin 3-sambubioside | 5.1 | **8.0** | 4.7 | **9.4** | 0.0 | **0.0** | 2.7 | **9.5** | 3.5 | **9.7** |
| | Pelargonidin 3-sophoroside / Cyanidin 3-rutinoside | 13.4 | **7.4** | 4.8 | **9.7** | 0.0 | **4.5** | 41.8 | **11.3** | 45.5 | **11.6** |
| | Cyanidin 3-sambubioside-5-rhamnoside | 0.9 | **6.7** | 1.2 | **9.3** | 0.0 | **0.0** | 0.9 | **10.5** | 1.8 | **11.4** |
| | Pelargonidin 3-sophoroside-5-rhamnoside | 0.8 | **6.2** | 0.0 | **1.8** | 0.0 | **0.0** | 6.4 | **12.2** | 6.3 | **12.2** |
| | Pelargonidin 3-glucoside | 2.1 | **6.1** | 0.6 | **5.7** | 0.0 | **0.0** | 3.7 | **7.4** | 1.8 | **6.9** |
| | Pelargonidin 3-sambubioside | 0.0 | **0.0** | 0.0 | **2.4** | 0.0 | **0.0** | 0.0 | **0.0** | 0.0 | **0.6** |
| | Pelargonidin 3-rutinoside | 0.0 | **0.0** | 0.0 | **0.0** | 0.0 | **0.0** | 0.0 | **0.0** | 0.0 | **0.0** |
| | **Total Anthocyanins** | 280.3 | **6.36** | 157.7 | **6.68** | 4.0 | **4.04** | 566.2 | **9.41** | 646.0 | **8.45** |
| **Flavonols** | Quercetin 3-glucosylrutinoside | 0.76 | **8.8** | 0.04 | **8.4** | 0.00 | **0.0** | 3.64 | **14.8** | 5.12 | **13.6** |
| | Quercetin diglucoside | 0.28 | **8.2** | 1.52 | **14.4** | 0.01 | **0.6** | 3.05 | **48.9** | 2.39 | **26.2** |
| | Quercetin 3-galactosylrhamnoside | 0.25 | **10.3** | 0.00 | **0.0** | 0.00 | **0.0** | 0.25 | **25.3** | 0.88 | **18.3** |
| | Quercetin 3-rutinoside | 0.31 | **9.3** | 0.05 | **7.9** | 0.01 | **56.9** | 0.50 | **16.8** | 0.64 | **16.0** |
| | Quercetin 3-galactoside | 0.97 | **3.0** | 0.42 | **6.6** | 0.07 | **0.0** | 0.95 | **8.3** | 1.91 | **7.1** |
| | Quercetin 3-glucuronide | 5.48 | **4.0** | 0.44 | **10.3** | 2.12 | **7.9** | 1.11 | **14.7** | 3.54 | **9.0** |
| | Quercetin 3-glucoside | 0.66 | **8.1** | 0.16 | **7.0** | 0.95 | **7.3** | 0.25 | **10.2** | 0.56 | **12.0** |
| | Quercetin HMGG | 0.01 | **2.6** | 0.00 | **0.0** | 0.00 | **0.0** | 0.00 | **0.0** | 1.57 | **9.4** |
| | Kaempferol 3-galactoside | 0.08 | **5.3** | 0.02 | **5.5** | 0.00 | **0.0** | 0.22 | **12.2** | 0.23 | **11.3** |
| | Kaempferol 3-glucuronide | 0.44 | **5.9** | 0.02 | **8.7** | 0.08 | **8.9** | 0.21 | **14.3** | 0.31 | **11.2** |
| | Kaempferol 3-glucoside | 0.11 | **8.4** | 0.01 | **8.4** | 0.03 | **8.7** | 0.07 | **11.8** | 0.16 | **13.2** |
| | **Total Flavonols** | 9.35 | **6.72** | 2.68 | **7.02** | 3.27 | **8..21** | 10.24 | **16.12** | 17.33 | **13.39** |
| **Ellagic acid derivatives** | Ellagic acid pentoside A | 1.23 | **10.1** | 0.95 | **11.2** | 3.53 | **15.1** | 4.48 | **18.4** | 5.51 | **15.7** |
| | Ellagic acid pentoside B | 1.35 | **10.1** | 1.89 | **10.3** | 2.51 | **15.5** | 3.52 | **15.8** | 0.61 | **13.6** |
| | Ellagic acid | 1.58 | **21.2** | 1.68 | **18.9** | 3.05 | **28.7** | 4.65 | **47.0** | 3.52 | **17.8** |
| | Methyl ellagic acid pentoside A | 0.70 | **17.4** | 0.52 | **15.3** | 2.49 | **21.4** | 1.86 | **22.0** | 4.56 | **26.8** |
| | Ellagic acid 4-acetylxyloside A | 0.44 | **6.6** | 0.43 | **7.4** | 0.38 | **6.5** | 0.34 | **8.1** | 0.35 | **7.9** |
| | Methyl ellagic acid pentoside B | 0.16 | **17.0** | 0.24 | **14.3** | 0.66 | **22.9** | 0.47 | **23.9** | 0.42 | **69.3** |
| | Ellagic acid 4-acetylarabinoside B | 1.05 | **7.2** | 1.54 | **7.5** | 0.65 | **10.2** | 0.78 | **11.4** | 0.34 | **18.7** |
| | **Total EA derivatives** | 6.51 | **12.8** | 7.25 | **12.13** | 13.27 | **17.19** | 16.10 | **20.94** | 15.30 | **24.25** |
| **Ellagitannins** | Lambertianin C | - | **0.07** | - | **0.03** | - | **0.01** | - | **0.49** | - | **0.08** |
| | Sanguiin H6 | - | **2.35** | - | **1.89** | - | **2.61** | - | **4.39** | - | **3.05** |
| | **Sum of % Ellagitannins*** | | **2.42** | | **1.92** | | **2.62** | | **4.88** | | **3.13** |

Recoveries are percentages of the contents in the original material (see Table S1). *-The ellagitannins are only expressed as % recovery values.

**Supplementary Table S3B. Identification and recovery of other polyphenols in raspberry GIB fractions**

| Compound | | | | 00123A7 | | 304F6 | | 2J19 | | Glen Ericht | | Tulameen | |
|---|---|---|---|---|---|---|---|---|---|---|---|---|---|
| | $R_t$ (min) | $[M+H]^+$ (*m/z*) | $MS^2$ (*m/z*) | µg $g^{-1}$ FDW | %Rec | µg $g^{-1}$ FDW | %Rec | µg $g^{-1}$ FDW | %Rec | µg $g^{-1}$ FDW | %Rec | µg $g^{-1}$ FDW | %Rec |
| **Phenolic acids (µg $g^{-1}$ FDW)** | | | | | | | | | | | | | |
| Hydroxybenzoic | 1.27 | 137.0233 | 93 | **0.15** | NA | **0.09** | NA | **0.17** | NA | **0.20** | NA | **0.11** | NA |
| Protocatechuic | 1.70 | 153.0180 | 109 | **0.10** | NA | **0.06** | NA | **0.01** | NA | **0.17** | NA | **0.19** | NA |
| Cinnamic | 3.60 | 147.0440 | - | **0.02** | NA | **0.01** | NA | **0.02** | NA | **0.03** | NA | **0.01** | NA |
| Caffeic | 3.92 | 179.0335 | 135 | **0.13** | NA | **0.15** | NA | **0.04** | NA | **0.17** | NA | **0.11** | NA |
| Coumaric | 5.45 | 163.0389 | 119 | **0.09** | NA | **0.04** | NA | **0.17** | NA | **0.14** | NA | **0.15** | NA |
| Ferulic | 6.58 | 193.0491 | 178, **149**, 134 | **0.08** | NA | **0.08** | NA | **0.08** | NA | **0.11** | NA | **0.07** | NA |
| Sinapic | 7.02 | 223.0593 | - | **0.03** | NA | **0.02** | NA | **0.00** | NA | **0.00** | NA | **0.03** | NA |
| **Ellagitannin degradation products** | | | | | | | | | | | | | |
| Lambertianin C - EA | 7.31 | 1250.5948* | 1867, 1567, **1099**, 935, 933, 633 | - | 0.19 | -- | 0.14 | - | 0.14 | - | 0.48 | - | 0.17 |
| Sanguiin H10 A | 6.20 | 783.0612* | **1265,** 933, 633, 301 | - | 0.41 | - | 0.63 | - | 0.86 | - | 0.98 | - | 0.94 |
| Sanguiin H10 B | 7.07 | 783.0615* | **1265,** 933, 633, 301 | - | 1.21 | - | 1.24 | - | 2.06 | - | 2.09 | - | 2.12 |
| Sanguiin H2 | 7.73 | 551.0345* | 935, **633**, 469 | - | 0.14 | - | 0.16 | - | 0.29 | - | 0.23 | - | 0.23 |
| Galloyl glucoside A | 1.36 | 331.0641 | **313**, 169 | -- | 0.01 | - | 0.00 | - | 0.01 | - | 0.01 | - | 0.00 |
| Galloyl glucoside B | 1.72 | 331.0640 | 169 | - | 0.01 | - | 0.00 | - | 0.02 | - | 0.01 | - | 0.01 |
| Sanguiin H10 - EA A | 2.57 | 632.0599* | **933**, 613, 331, 301 | - | 0.01 | - | 0.01 | - | 0.02 | - | 0.02 | - | 0.02 |
| Sanguiin H10 - EA B | 3.24 | 632.0607* | **933**, 613, 331, **301** | - | 0.04 | - | 0.06 | - | 0.13 | - | 0.06 | - | 0.11 |
| Sanguiin H10 - EA C | 3.60 | 632.0603* | **933**, 613, 331, **301** | - | 0.01 | - | 0.01 | - | 0.03 | - | 0.01 | - | 0.03 |
| Sanguiin H10 - EA D | 3.99 | 632.0602* | 963, 933, 613**, 301** | -- | 0.01 | - | 0.03 | - | 0.05 | - | 0.03 | - | 0.05 |
| Sanguiin H10 - EA E | 6.58 | 632.0604* | 935, 933, **551**, 331, 301 | - | 0.02 | - | 0.04 | - | 0.05 | - | 0.04 | - | 0.05 |

Relative levels of phenolic acids assume all phenolic acids have similar ionisation responses.
NA - % recovery not possible as phenolic acids were not quantified in the original samples
Levels of Lambertianin C-ellagic acid derivatives are relative to Lambertianin C peak areas in the original sample assuming similar ionisation responses.
Levels of all other components are relative to Sanguiin H6 assuming similar ionisation responses. * denotes doubly charged ion.
